# Pregnancy and Lactation Alter Vitamin A Metabolism and Kinetics in Rats under Vitamin A-Adequate Dietary Conditions

**DOI:** 10.3390/nu13082853

**Published:** 2021-08-19

**Authors:** Yaqi Li, Ayasa Tajima, Floyd J. Mattie, Michael H. Green, A. Catharine Ross

**Affiliations:** Department of Nutritional Sciences, The Pennsylvania State University, University Park, PA 16802, USA; yxl277@psu.edu (Y.L.); Ayasa.tajima@gmail.com (A.T.); Fjm1311@gmail.com (F.J.M.); Mhg@psu.edu (M.H.G.)

**Keywords:** vitamin A, model-based compartmental analysis, pregnancy and lactation, metabolism and kinetics, dietary intake

## Abstract

Background: Vitamin A (VA) plays critical roles in prenatal and postnatal development; however, limited information is available regarding maternal VA metabolism during pregnancy and lactation. Objectives: We investigated the impact of pregnancy and lactation on VA metabolism and kinetics in rats, hypothesizing that changes in physiological status would naturally perturb whole-body VA kinetics. Methods: Eight-week old female rats (*n* = 10) fed an AIN-93G diet received an oral tracer dose of ^3^H-labeled retinol to initiate the kinetic study. On d 21 after dosing, six female rats were mated. Serial blood samples were collected from each female rat at selected times after dose administration until d 14 of lactation. Model-based compartmental analysis was applied to the plasma tracer data to develop VA kinetic models. Results: Our compartmental model revealed that pregnancy resulted in a gradual increase in hepatic VA mobilization, presumably to support different stages of fetal development. Additionally, the model indicates that during lactation, VA derived from dietary intake was the primary source of VA delivered to the mammary gland for milk VA secretion. Conclusion: During pregnancy and lactation in rats with an adequate VA intake and previous VA storage, the internal redistribution of VA and increased uptake from diet supported the maintenance of VA homeostasis.

## 1. Introduction

The critical roles of vitamin A (VA) in mammalian reproduction and embryonic development have been known for decades [1]. Numerous studies have revealed that VA is involved in multiple fetal organogenesis processes, and that either a deficiency or an excessive intake of VA is detrimental to fetal development [2,3,4,5,6,7,8,9,10,11]. Previous studies have investigated the VA content of the maternal liver, certain peripheral tissues, placenta, and embryo [12,13,14], while pharmacokinetic studies have focused on maternal VA kinetics and the exposure of the embryo to VA after dosing at selected gestational stages [15,16]. However, to date, no studies have investigated the dynamic changes in maternal VA metabolism that may occur across the period from pregnancy and lactation, from the viewpoint of whole-body retinol kinetics and compartmental analysis.

The combination of radioactive isotope labeled tracer kinetics and compartmental modeling has become a useful and powerful tool to study VA metabolism under different conditions of nutritional and metabolic stresses, such as dietary deficiency, inflammation, toxicant exposure, or at different life stages [17,18,19,20,21,22,23,24,25,26]. These studies have revealed that the kinetic behavior of VA varies with physiological status, and suggest that adaptive changes in retinol kinetics serve as a mechanism to maintain plasma retinol homeostasis under a variety of stressful conditions. However, no comprehensive investigation of changes in retinol kinetics during the normal physiological processes of pregnancy and lactation has yet been reported. Relatively little is known about maternal VA metabolism during pregnancy and lactation, even though maternal VA status is directly associated with birth outcomes [1,7,8,9]. Therefore, in the current study, we applied model-based compartmental analysis and tracer kinetic techniques to explore VA kinetics during pregnancy and lactation in the rat as a well-studied animal model. Because of the limited knowledge about VA metabolism during these physiological periods, we began with the general hypotheses that VA kinetics would be perturbed during pregnancy and lactation and that model-based compartmental analysis would help reveal potential changes in VA trafficking during these physiological transitions. Such results are expected to enhance knowledge about VA kinetics during pregnancy and lactation, set the foundation for future investigation of the impact of nutritional status on VA metabolism during these life stages, and provide insights for future dietary recommendations.

## 2. Materials and Methods

### 2.1. Animals and Diets

Thirty-eight Sprague Dawley rats (7-week-old females (*n* = 27) and 9-week-old males (*n* = 11)) were purchased from Charles River Laboratory (Wilmington, MA, USA). Upon arrival and throughout the study, rats were fed a standard AIN-93G diet (Research Diets, New Brunswick, NJ, USA) with free access to food and water. Diet intake was measured every 2–3 days. Rats were housed individually in solid plastic cages with bedding and nesting material in an environmentally controlled animal facility. All animal protocols were approved by the Institutional Animal Care and Use Committee of The Pennsylvania State University.

### 2.2. Preparation of 11,12-^3^H-Retinol-Labeled Oral Dose

As modified from a method described previously [19,27], a known amount of 11,12-^3^H-retinol (Perkin-Elmer, Waltham, MA, USA) was added to a carrier amount of unlabeled retinol, then solubilized in canola oil, so that radioactivity was estimated to be ~0.4 μCi/μL of dose. The radioactive dose was prepared fresh and vortexed before administration.

### 2.3. Kinetic Studies

**Figure 1:**
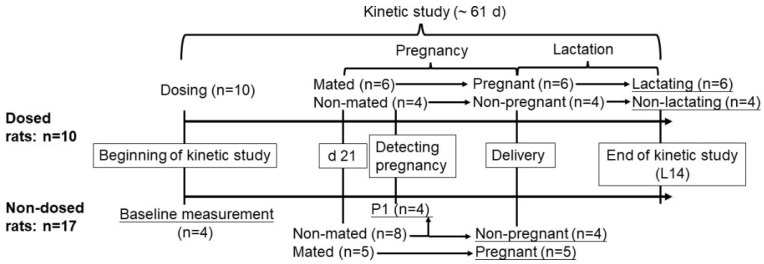
Study design for the VA kinetic study. The upper arrow represents the timeline for the dosed rats (*n* = 10), and the lower arrow represents the timeline for non-dosed rats (*n* = 17). Underlines indicate the time of euthanasia. P1, pregnancy day 1; L14, lactation day 14.

An overview of the study design is shown in Figure 1. After 1 week of acclimation, *n* = 10 female rats received an accurately measured volume (15 μL) of the 11,12-^3^H-retinol-labeled dose orally by micropipette to initiate the kinetic study; the pipette tip used for dosing and the chip used for wiping the un-ingested dose from the animal’s mouth were collected for counting; details of dose administration were described previously [19]. Serial blood samples (*n* = 14) were collected from a caudal vein at selected times from 2 h to 21 d after dose administration. On d 21, six of the dosed females were housed with a male rat for a week for mating; these rats are referred to hereafter as the pregnant–lactating group. Vaginal plugs were checked daily to determine d 1 of pregnancy (P1), and were found in 2 of the 6 rats; for the 4 rats for which no vaginal plug was found, P1 was determined by back-counting 21 days from the day of parturition. The bleeding schedule for the 6 females in the pregnant–lactating group was adjusted individually from P1 at a frequency of every 2–4 days until the time of delivery, while the four non-mated females (referred to as the non-pregnant comparison group) were bled every 4 days. For the 6 rats in the pregnant–lactating group, the date of delivery was considered d 1 of lactation (L1); litter size was normalized to ten pups/dam and blood collection for both the pregnant–lactating group and the non-mated comparison group was continued until d 14 of lactation (L14) with an interval of 2–3 days between each collection. At the end of the study, all rats were euthanized by carbon dioxide inhalation, livers were collected and stored at −80 °C, and plasma was separated and stored at −20 °C until analysis.

In parallel to the rats for which serial blood samples were collected throughout the kinetic study period, an age-matched group of non-dosed rats was fed an identical diet and rats from this group were euthanized at certain times to determine the plasma and liver VA status corresponding to the different physiological states in the dosed pregnant–lactating rats. Briefly, four female rats were euthanized at the beginning of the kinetic study (baseline), four were euthanized at the time that pregnancy was detected (detection of pregnancy), and five pregnant (mated at the same time as the dosed rats) and four non-pregnant rats were euthanized at the time of delivery (representing the end of pregnancy/beginning of lactation).

### 2.4. VA Mass Quantification

Plasma and liver samples collected at euthanasia were analyzed using ultra-performance liquid chromatography (UPLC) to determine VA mass as previously described [28]. For each plasma sample, an aliquot of 100 µL was used; for liver, a weighed portion of ~0.05 g frozen sample was taken for analysis.

### 2.5. Radioactivity Measurement

As described previously [25], plasma (35–300 μL) was separated from each blood sample after centrifugation and transferred to a glass vial preloaded with scintillation fluid (Fisher Chemical, Waltham, MA, USA). The tracer concentration in the plasma and dose ^3^H content was then measured by liquid scintillation spectrometry (Beckman Coulter, Brea, CA, USA) to a 2-sigma error of 1% or for a maximum of 300 min.

### 2.6. Kinetic Data Calculations

The total radioactivity (^3^H dpm) administered to each rat was calculated based on the measured tracer concentration in the dose × volume administered; a correction was applied based on the radioactivity remaining in the pipette tips and chips used for oral dosing (see [19] for detailed procedures). Then, the plasma tracer amount at each sampling time was calculated from the measured tracer concentration × estimated plasma volume, where plasma volume was estimated as 0.035 mL/g body weight [19], and body weight was measured each time at sample collection. By dividing the tracer content in plasma by the total dose received by each rat, we determined the fraction of the dose in plasma (FDp) at each time. Since the bleeding times were the same among non-mated comparison group rats, the geometric mean of FDp at each sampling time was calculated and used in mathematical modeling for the group of non-mated females, while the data for rats in the pregnant–lactating group were modeled individually before averaging for the group.

### 2.7. Model Development and Kinetic Parameters

**Figure 2:**
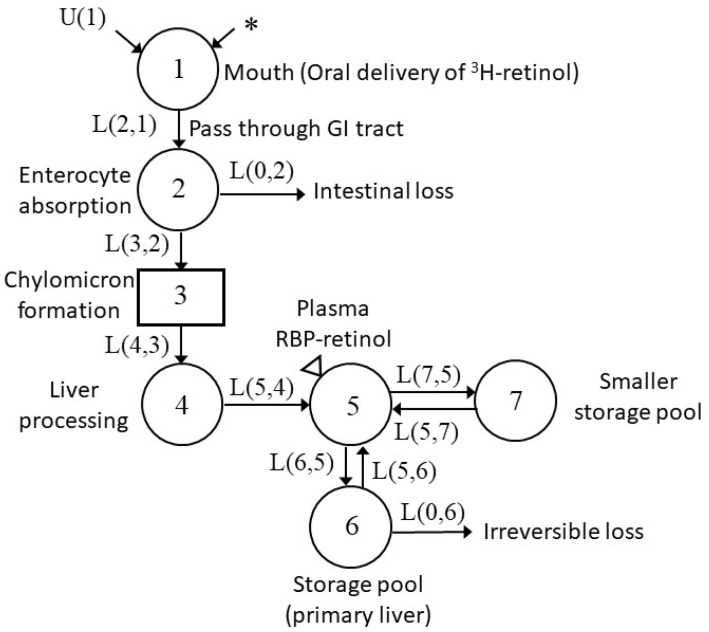
Conceptual model for VA tracer kinetics in rats. Each circle represents a different compartment, the rectangle represents a delay component. Arrows indicate the transfer of VA from one compartment to another compartment, where, for example, L(5,4) represents the fraction of VA in compartment 4 that is transferred to compartment 5 (plasma) per day. The asterisk indicates the site of the ^3^H-labeled retinol dose entering the system, U(1) indicates the dietary intake of VA, and the triangle indicates the site of sample collection (plasma).

Using the Windows version of the Simulation, Analysis and Modeling software (WinSAAM; [29]), we applied compartmental modeling to analyze the plasma tracer data. As a starting point, we adapted a previously published physiological model (Figure 2) that characterized VA kinetics after oral dosing [19]. Then, the initial conditions, initial estimates of the fractional transfer coefficients (L(I,J)s (see [29]), the fraction of VA in compartment J that is transferred to compartment I each day), and the FDp (group geometric mean FDp) for non-pregnant comparison group rats and FDp values for individual pregnant–lactating rats at each sampling time, with a fractional standard deviation (FSD) of 0.1 as the weighting factor, were entered into a WinSAAM input file. By iteratively adjusting the model parameters and structure, and adding time-interrupts TC(I), which is an embedded function in the WinSAAM software allowing us to change the value of parameters at targeted times to reflect changes in physiological state that could potentially perturb the system [25,29], we obtained a best fit between the observed data and model-calculated values (i.e., when no improvement could be observed in the fitting). Then, final values for the L(I,J)s and their statistical uncertainties were obtained using weighted nonlinear regression analysis in WinSAAM, and other kinetic parameters of interest, such as R(I,J) (VA mass transferred from compartment J to compartment I each day), were calculated (see Figures 5–7 for compartment identification). Further details of the model development process are described in the Results section.

### 2.8. Statistical Analysis

Data are reported as means with SD. Differences were determined by an unpaired *t*-test. All statistical tests were performed using GraphPad Prism 9.0. A *p* value < 0.05 was considered statistically significant.

## 3. Results

### 3.1. Physiological Outcomes

Unless specifically mentioned, all the results reported are from the ^3^H-VA dosed rats (*n* = 10) in the kinetic study, six in the pregnant–lactating group and four in the non-mated comparison group. All of the mated rats (*n* = 6) became pregnant and delivered pups. The diet records (Table 1) showed that before pregnancy, neither daily diet nor VA intake differed between the non-pregnant comparison group and the pregnant–lactating group. However, after that, pregnant females consumed nearly 30% more food compared to non-pregnant rats (*p* < 0.01 for both diet intake and VA intake). Furthermore, during lactation, the consumption of food and intake of VA more than doubled compared to the non-pregnant rats (*p* < 0.0001 for both diet and VA intake).

### 3.2. Changes in VA Tissue Status during Pregnancy and Lactation

Our study included a parallel group of rats matched for age and physiological status that were not dosed with ^3^H-VA, along with the dosed rats, to determine VA amounts in plasma and liver at the beginning of pregnancy, at delivery, and at the end of lactation (Table 2). Compared to non-pregnant rats, plasma VA mass was higher in pregnant–lactating rats at the end of pregnancy (*p* < 0.001), whereas liver VA mass trended lower but did not differ significantly (*p* = 0.27). When comparing VA status at different stages in the pregnant–lactating rats, plasma VA mass significantly increased throughout pregnancy (*p* < 0.001), and went back to the pre-pregnancy level at the end of lactation (*p* < 0.01).

### 3.3. Development of Compartmental Model for VA Kinetics in Non-Pregnant Rats

**Figure 3:**
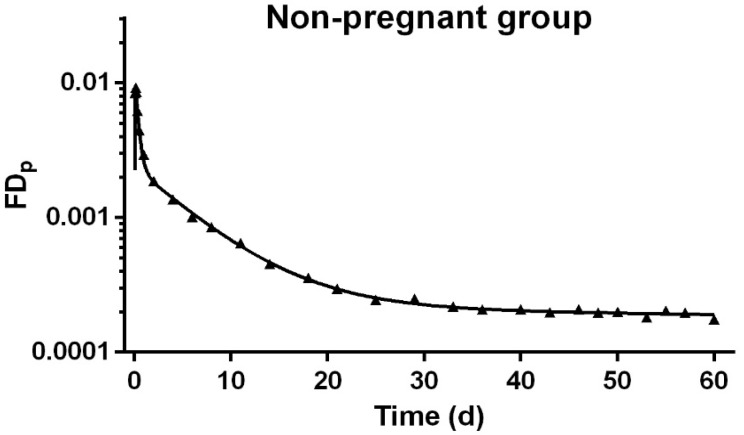
Fraction of administrated dose in plasma (FDp) versus time (d) after oral delivery of ^3^H-labeled retinol in non-pregnant rats. Solid triangles are observed data presented as group geometric mean, *n* = 4; the line represents the model-predicted value.

Based on previous work [19], we began our modeling with a seven-compartment model (Figure 2) to describe VA kinetics in the non-pregnant comparison group. This model included compartments representing the digestion and absorption of the oral dose and processing of absorbed VA in the liver (compartments 1–4); the secretion of retinol-binding protein (RBP)-bound retinol from hepatocytes to plasma, and the exchange of VA between plasma (compartment 5) and two extravascular pools (compartment 6 representing a slowly turning-over storage pool and compartment 7 representing a smaller but more rapidly turning-over storage pool). By adjusting the kinetic parameters, we obtained a good fit to the observed geometric mean data for the group of four non-pregnant reference rats (Figure 3). Parameters of interest and their statistical uncertainties are summarized in Table 3. All parameters were well identified, with FSD ≤ 0.2.

### 3.4. Development of Compartmental Model for VA Kinetics in Pregnant–Lactating Rats

**Figure 4:**
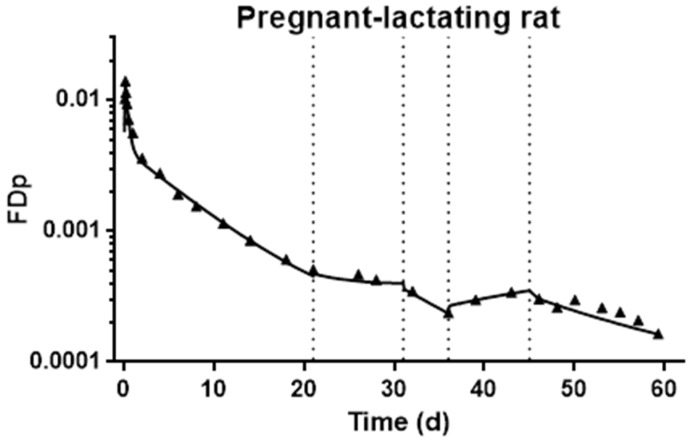
Fraction of administrated dose in plasma (FD_p_) versus time (d) after oral delivery of ^3^H-labeled retinol in one representative pregnant–lactating rat. Solid triangles are observed data; the solid line represents the model-predicted fit; the dashed line indicates the times of perturbation introduced in modeling: the first dashed line (d 21) represents the beginning of mating, the second dashed line (d 31) represents early pregnancy, the third dashed line (d 36) represents late pregnancy, and the fourth dashed line (d 45) represents the beginning of lactation.

Firstly, immediate visual inspection of the plasma ^3^H tracer response curves showed differences in the pregnant–lactating rats, compared to the non-mated comparison group, most notably as abrupt transitions which signaled that modeling these data would require adaptations that were not required for modeling the non-pregnant comparison group. During the kinetic study, rats in the pregnant–lactating group underwent several physiological stages: before mating, mating, pregnancy, and lactation. Therefore, to characterize and compare the potential differences in VA kinetics at these stages, the kinetic model we developed for the group of non-pregnant reference rats was modified sequentially to reflect the physiological changes that occurred during pregnancy and lactation. As described in the subsequent paragraphs, we developed these sequential models for one representative rat from the pregnant–lactating group and then applied the same strategies to model the data for the other five rats in the group. The tracer response curve for a representative pregnant–lactating rat over 60 d is shown in Figure 4. During the first 21 d after dose administration, before mating, we used the model in Figure 2 and estimates for parameters that were obtained for the non-pregnant group to arrive at a satisfactory fit for tracer data for the pregnant–lactating rats (Figure 4). The mean values for the kinetic parameters during this first non-pregnant period are listed in Table 3. The differences in the L(I,J) values were within the acceptable range of individual variance, as no difference in kinetic curves was observed between non-pregnant and pregnant–lactating groups by visual inspection. As expected, there were no statistically significant differences in kinetic parameters for the two groups, as they were physiologically identical, providing evidence that rats in the two groups had similar VA kinetics before the initiation of mating.

**Figure 5:**
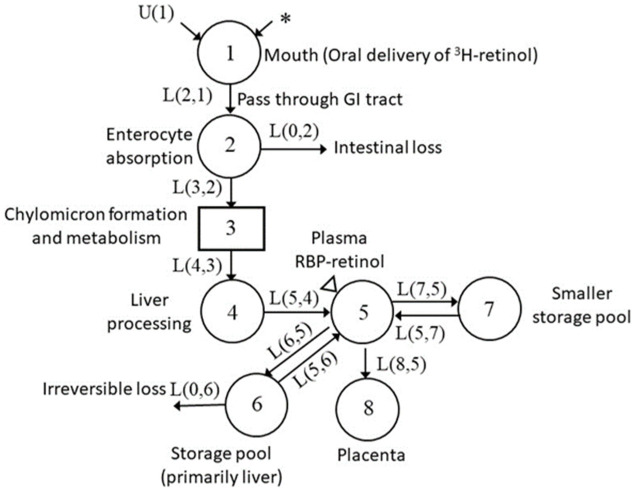
Proposed model for VA tracer kinetics in pregnant–lactating rats in early pregnancy. Each circle represents a different compartment, the rectangle represents a delay component, arrows indicate the transfer of VA from one compartment to another compartment. The asterisk indicates the site of entry of the ^3^H-labeled retinol dose into the system, U(1) indicates the dietary intake of VA, and the triangle indicates the site of sample collection (plasma).

Next, we then fixed parameters obtained during the pre-mating stage and we introduced a time-interrupt, TC(1), at d 21, representing the beginning of mating. To obtain a good fit to these ^3^H tracer data, we decreased dietary VA input and increased VA mobilization from the slowly turning-over storage pool to plasma. At d 31, a second time-interrupt, TC(2), was introduced corresponding to the observed decrease in plasma tracer, and we defined the period immediately following as early pregnancy. During this stage, we added an additional compartment to the existing physiological model to represent the placenta (Figure 5) and account for the transfer of VA from plasma to placenta. Obtaining a good fit for the data also required a decrease in VA secretion from the slowly turning-over storage pool and an increase in dietary VA input, which we estimated using the dietary record for each rat.

**Figure 6:**
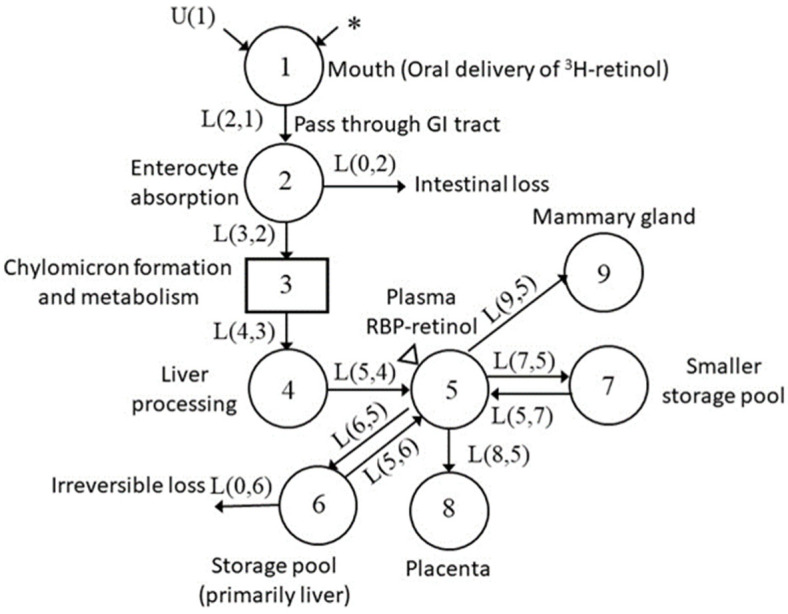
Proposed model for VA tracer kinetics in pregnant–lactating rats at late pregnancy. Each circle represents different compartment, the rectangle represents a delay component, arrows indicate the transfer of VA from one compartment to another compartment. The asterisk indicates the site of the ^3^H-labeled retinol dose entering the system, U(1) indicates the dietary intake of VA, and the triangle indicates the site of sample collection (plasma).

At d 36, plasma tracer started to increase, suggesting a switch to a new kinetic stage and implying that there must be a transfer of ^3^H-retinol from an exchangeable pool into plasma. Accordingly, we implemented a third time-interrupt, TC(3). During this period, a compartment representing the mammary gland was added to the model (Figure 6). Dietary input was the same as in the early pregnancy stage, while the mobilization of VA from the slowly turning-over storage pool was increased to drive the observed increase in plasma ^3^H tracer and support the transfer of VA from the plasma pool to the placenta and mammary gland.

**Figure 7:**
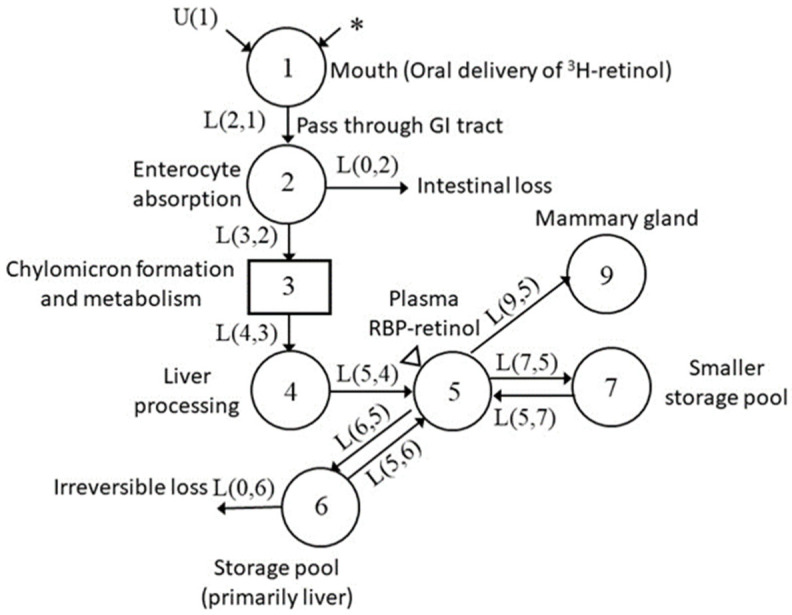
Proposed model for VA tracer kinetics in pregnant–lactating rats during lactation. Each circle represents a different compartment, the rectangle represents a delay component, arrows indicate the transfer of VA from one compartment to another compartment. The asterisk indicates ^3^H-labeled retinol dose entering the system, U(1) indicates the dietary intake of VA, and the triangle indicates the site of sample collection.

After parturition and with the initiation of lactation (d 45), the model structure (Figure 7) was modified again, this time with the removal of the placenta compartment, and introduction of a final time-interrupt, TC(4). Dietary input was further increased compared to the pregnancy stage, along with the transfer of plasma VA to the mammary gland, whereas mobilization from the VA slowly turning-over storage pool to plasma was decreased. With the completion of fitting data for the lactation period, we obtained a good fit of plasma tracer data throughout the entire kinetic study (see Figure 4). After completing the model for the representative rat, we then applied the same modeling strategy to the data for each of the other pregnant–lactating rats (see Supplementary Figure S1 for individual kinetic curves). After reaching a satisfactory fit for each individual rat, we then calculated and summarized the group mean of the kinetic parameters that represented the physiological changes during the different physiological stages (Table 4). In agreement with what we observed in the representative rat, we found that during mating, the group mean for the model-estimated dietary input (U(I)) was decreased, whereas the daily hepatic secretion of VA (R(5,6)) was increased. In early pregnancy, increased dietary VA intake was able to support plasma VA transfer to placenta, thus less hepatic VA secretion was required. When entering late pregnancy, hepatic mobilization was activated as a result of increased plasma VA transfer to the placenta and the initiation of VA deposition in the mammary gland. During lactation, dietary VA input was further increased, consistent with dietary records, as well as the transfer of plasma VA to the mammary gland [30], while liver VA secretion was reduced.

## 4. Discussion

By combining tracer kinetic techniques and model-based compartmental analysis, we studied VA metabolism and kinetics in non-pregnant and pregnant–lactating rats and developed compartmental models to describe the physiological changes during pregnancy and lactation. As shown in Figure 3 and Figure 4, we obtained satisfactory fits to the tracer data for non-pregnant and pregnant–lactating rats, respectively. While no perturbation occurred in the non-pregnant rats, four perturbations were apparent in the pregnant–lactating group, representing the initiation of mating, early pregnancy, late pregnancy, and lactation, and thus we divided the VA kinetics of the pregnant–lactating group into five stages.

In the first stage, before any mating events occurred, no difference was observed in the VA kinetic behaviors between the groups of non-pregnant and pregnant–lactating rats, as expected and as supported by the kinetic parameters shown in Table 3, as no statistical differences were found between the two groups, as well as no difference in dietary VA intake (Table 1), thereby indicating that under the same housing and dietary condition, rats in the two groups behaved the same in terms of VA kinetics. These data also suggest that the potential future differences in plasma ^3^H kinetics between the two groups could be reliably attributed to pregnancy and lactation.

When the mating began, represented as TC(1) in our model, it was not possible to measure the actual diet consumption of the mating females because the male and female rats were kept in the same cage; however, a nearly 80% reduction in dietary input (UF(1)) was needed in the compartmental model to fit the plasma data; in addition, an increase to 160% of the prior value in the liver VA mobilization to plasma (R(5,6)) was required (Table 4). These two kinetic parameters identified from modeling suggested that introducing the male rats into the cages of the females may have caused stress in the female rats, affecting their appetite and reducing their food intake, and further suggests that to compensate for the decrease in VA intake and maintain whole-body VA status, VA was mobilized from liver stores during this time. The model-predicted increase in hepatic VA secretion was in response to the decreased dietary VA intake, indicating a central regulatory role of the liver in whole-body VA homeostasis, in agreement with well-established theories of VA homeostasis revealed in previous studies [29,31,32,33]. According to our model, this alteration in status lasted from d 21 to d 31, covering the period of mating and very early stages of pregnancy (estimating that the date of conception occurred on d 25), as evidenced by previous findings that no maternal VA transfer to the conceptus occurred at this time [12,14]. On the other hand, pregnant female rats showed a relatively elevated and steady concentration of plasma VA (Figure 4) at this stage, suggesting a self-adaptive mechanism in the pregnant animal to guarantee the availability of plasma VA, in preparation for the later transfer of VA to the placenta.

A second time-interrupt TC(2) signaled the period we have called early pregnancy in our study, at which time maternal VA transfer to the placenta was initiated. From our modeling results, in this period, the observed decrease in plasma tracer was attributed to increased dietary VA intake, decreased VA output from liver, and the initial transfer of VA from maternal plasma to the placenta. Dietary records indicated that rats ingested more VA at this time (Table 1), and we used this information as a modeling input. Therefore, the model-predicted dietary intake was nearly 17% higher compared to that before pregnancy (Table 4). At the same time, the hepatic secretion of VA was downregulated in response to the increased dietary input (Table 4). Additionally, more importantly, at this stage, we added the additional compartment 8 to represent the early transfer of maternal VA to the placenta. As indicated by the fractional transfer coefficient L(8,5) in Table 3, there was only limited transfer of VA at this phase, confirming previous findings based on VA mass analysis [12,14].

A third time-interrupt TC(3) was required in the modeling process (around P11), referred to here as late pregnancy. This time-interrupt aligned physiologically with the time when VA is required for fetal growth and organogenesis [5]. With the increased need for VA to support fetal development, the transfer of maternal VA to placenta further increased (Table 4; L(8,5) increased from 0.463 to 0.724 per day), as suggested previously by the increased VA accumulation in the placenta and embryo, as well as the detection of RBP in fetal microsomes at this stage [12,14,34]. Meanwhile, VA transfer to the mammary gland began (L(9,5) in Figure 7 and Table 4), with L(9,5) increasing from 0.0108 to 0.0844 per day between early and late pregnancy, to prepare for milk production after delivery. With the increased demand for plasma VA output, as well as the decreased plasma VA from early pregnancy, the hepatic mobilization of VA was apparently activated (Table 4), and this increased export of VA from the slowly turning-over storage pool could serve as the driving force leading to the observed increase in plasma tracer at the late pregnancy stage. The early decline and later climb in plasma ^3^H tracer was universal among all rats in the pregnant–lactating group (Supplementary Figure S1) and was similar to that observed by Satre et al. as well [14]. While an increased hepatic mobilization of VA was identified by the model, by comparing the measured VA mass at the beginning and end of pregnancy in the parallel group of rats from which tissues were collected (Table 2), it can be seen that the liver VA mass showed a tendency to decrease during pregnancy, while plasma VA mass was significantly elevated. Together, these results suggest that the liver was the major source of VA to support the increase in plasma VA.

During lactation, the fifth stage in our analysis, the placental compartment no longer existed, but the plasma VA transfer to the mammary gland was elevated with the onset of milk production (Table 4). In addition, dietary intake was almost doubled during this period (Table 1), which was corroborated by the greatly increased dietary input based on the model prediction (Table 4). On the contrary, liver secretion of VA was reduced to less than half of its pre-mating value, which was supported by the relatively increased liver VA mass at the end of lactation (Table 2). Increased dietary input and decreased liver mobilization confirmed previous findings that dietary VA rather than body VA storage was the primary source of VA for mammary gland milk production, at least under conditions of a VA-sufficient diet [30,35,36,37].

## 5. Conclusions

In conclusion, with the application of model-based compartmental analysis to plasma retinol tracer kinetics, we developed a compartmental model that accounts for changes during pregnancy and the majority of the lactation period, and we mapped the changes in model-derived kinetic parameters to the corresponding physiological processes. Our results distinguish between different phases in maternal VA kinetics during pregnancy/lactation and identify the primary sources of VA input into plasma (dietary intake and/or liver VA stores) that support the maternal transfer of VA to the growing fetus at different gestational stages. Our results indicate that dietary VA is the major contributor to support the demanding VA transfer from plasma to the mammary gland during lactation and that the lactating rat was able to spare hepatic VA during this period. These findings highlight and reinforce the predominant role of the liver in regulating whole-body VA metabolism during pregnancy and lactation and they indicate that, in the well-nourished state and with adequate maternal VA storage, no VA supplementation is needed to support the increased VA requirement during pregnancy and lactation.

Assuming that our results in an animal model have translational value to humans, the results suggest that a VA-adequate female is able to meet the needs of the pregnancy–lactation period through a variety of internal adaptations, where internal VA stores are mobilized during pregnancy and diet assumes an important role in maintaining VA homeostasis during lactation. A corollary may be that additional VA from supplements would not be needed during this period, as the VA storage pools and mother’s consumption of VA from a usual VA-adequate diet appear to be sufficient for the VA needs of the mother-infant dyad.

## Figures and Tables

**Table 1:** Average intakes of diet and VA at different stages for non-pregnant and pregnant–lactating rats ^1^.

Period of Study	Intake	Non-Pregnant Group	Pregnant–Lactating Group
Before mating	Diet, g/d	19.7 (2.69)	20.0 (2.67)
	VA, nmol/d	82.6 (11.3)	83.8 (11.2)
During pregnancy	Diet, g/d	18.9 (2.23)	24.5 (2.85) *
	VA, nmol/d	79.0 (9.35)	103 (11.9) *
During lactation	Diet, g/d	18.1 (3.23)	41.4 (2.59) *
	VA, nmol/d	76.0 (13.5)	173 (10.9) *

^1^ Values are means with SD in parentheses. *n* = 4 for non-pregnant group, *n* = 6 for pregnant–lactating group. * indicates statistical difference between groups, *p* < 0.05.

**Table 2:** Plasma and liver VA mass at different times for non-pregnant and pregnant–lactating group rats ^1^.

Time of Study	VA Mass	Non-Pregnant Group	Pregnant–Lactating Group
Detection of pregnancy	Plasma, nmol	9.38 (1.51) ^a^	
	Liver, nmol	5289 (376)	
Parturition	Plasma, nmol	10.4 (1.60)	14.6 (0.586) *^b^
	Liver, nmol	5715 (835)	4702 (1507)
End of lactation	Plasma, nmol	11.6 (3.60)	11.3 (1.74) ^a^
	Liver, nmol	5726 (1004)	5503 (912)

^1^ Values are means with SD in parentheses, *n* = 4 for non-pregnant group, *n* = 5–6 for pregnant–lactating group, except for the measures at detection of pregnancy, when 4 non-pregnant rats were used to represent both groups. * indicates statistically significant difference between non-pregnant and pregnant–lactating groups; letters indicate statistically significant difference within pregnant–lactating group, *p* < 0.05.

**Table 3:** Fractional transfer coefficients for the 7-compartment models of VA metabolism showing no difference between non-pregnant and pregnant–lactating rats prior to mating ^1^.

	Model Estimates (FSD)	
L(I,J) ^2^	Non-Pregnant Group (Day^−1^)	Pregnant–lactating Group (Day^−1^)
L(5,4)	3.04 (0.09)	3.05 (0.1)
L(6,5)	8.01 (0.1)	4.33 (0.04)
L(5,6)	0.0117 (0.04)	0.00558 (0.09)
L(0,6)	0.00237 (0.2)	0.00251 (0.1)
L(7,5)	33.0 (0.1)	22.8 (0.1)
L(5,7)	0.744 (0.08)	0.895 (0.1)

^1^ Data shown here are model-derived values for each parameter with model-estimated FSDs in parentheses, *n* = 4 for the non-pregnant group, *n* = 6 for the pregnant–lactating group. FSD, fractional SD. ^2^ L(I,J)s represent the fraction of VA in compartment J that is transferred to compartment I each day.

**Table 4:** Kinetic parameters at different physiological stages for rats in the pregnant–lactating group ^1^.

Parameters	Before Mating	Mating	Early Pregnancy	Late Pregnancy	Lactation
UF(1), nmol/day ^2^	80.2 (9.74)	17.2 (27.9)	93.7 (5.99)	93.7 (5.99)	157 (10.4)
R(5,6), nmol/day ^3^	27.7 (5.40)	44.7 (8.69)	18.9 (7.58)	57.2 (11.1)	11.7 (7.42)
L(8,5) ^4^, day^−1^	0	0	0.463 (0.180)	0.724 (0.388)	0
L(9,5), day^−1^	0	0	0	0.0108 (0.0)	0.0844 (0.132)

^1^ Values are means with SD in parentheses, *n* = 6; ^2^ UF(1), model predicted dietary input; ^3^ R(I,J), VA mass transferred from compartment J to compartment I each day (see Figure 5, Figure 6 and Figure 7 for compartment identification); ^4^ L(I,J), fraction of VA in compartment J that is transferred to compartment I each day.

## Data Availability

The data that support the findings of this study are available from the corresponding author upon reasonable request.

## Supplementary Materials

The following are available online at https://www.mdpi.com/article/10.3390/nu13082853/s1, Figure S1: fraction of administrated dose in plasma versus time (d) after oral delivery of ^3^H-labeled retinol in pregnant–lactating rats.

## References

[1] Ross A.C., Gardner E.M. (1994). The function of vitamin A in cellular growth and differentiation, and its roles during pregnancy and lactation. Adv. Exp. Med. Biol..

[2] Warkany J., Schraffenberger E. (1946). Congenital malformations induced in rats by maternal vitamin A deficiency; defects of the eye. Arch. Ophthal..

[3] Wilson J.G., Warkany J. (1947). Anomalies of the genito-urinary tract induced by maternal vitamin A deficiency in fetal rats. Anat. Rec..

[4] Cohlan S.Q. (1953). Excessive intake of vitamin A as a cause of congenital anomalies in the rat. Science.

[5] Wilson J.G., Roth C.B., Warkany J. (1953). An analysis of the syndrome of malformations induced by maternal vitamin A deficiency. Effects of restoration of vitamin A at various times during gestation. Am. J. Anat..

[6] Nolen G.A. (1969). Variations in teratogenic response to hypervitaminosis A in three strains of the albino rat. Food Cosmet. Toxicol..

[7] Takahashi Y.I., Smith J.E., Winick M., Goodman D.S. (1975). Vitam A deficiency and fetal growth and development in the rat. J. Nutr..

[8] Geelen J.A. (1979). Hypervitaminosis A induced teratogenesis. CRC Crit. Rev. Toxicol..

[9] Rothman K.J., Moore L.L., Singer M.R., Nguyen U.S., Mannino S., Milunsky A. (1995). Teratogenicity of high vitamin A intake. N. Engl. J. Med..

[10] White J.C., Shankar V.N., Highland M., Epstein M.L., DeLuca H.F., Clagett-Dame M. (1998). Defects in embryonic hindbrain development and fetal resorption resulting from vitamin A deficiency in the rat are prevented by feeding pharmacological levels of all-trans-retinoic acid. Proc. Natl. Acad. Sci. USA.

[11] Antipatis C., Grant G., Ashworth C.J. (2000). Moderate maternal vitamin A deficiency affects perinatal organ growth and development in rats. Br. J. Nutr..

[12] Takahashi Y.I., Smith J.E., Goodman D.S. (1977). Vitamin A and retinol-binding protein metabolism during fetal development in the rat. Am. J. Physiol..

[13] Wallingford J.C., Underwood B.A. (1987). Vitamin A status needed to maintain vitamin A concentrations in nonhepatic tissues of the pregnant rat. J. Nutr..

[14] Satre M.A., Ugen K.E., Kochhar D.M. (1992). Developmental changes in endogenous retinoids during pregnancy and embryogenesis in the mouse. Biol. Reprod..

[15] Hummler H., Hendrickx A.G., Nau H. (1994). Maternal toxicokinetics, metabolism, and embryo exposure following a teratogenic dosing regimen with 13-cis-retinoic acid (isotretinoin) in the cynomolgus monkey. Teratology.

[16] Kang H.G., Ku H.O., Jeong S.H., Cho J.H., Son S.W. (2010). Evaluation of embryotoxic potential of olaquindox and vitamin a in micromass culture and in rats. Toxicol. Res..

[17] Lewis K.C., Green M.H., Underwood B.A. (1981). Vitamin A turnover in rats as influenced by vitamin A status. J. Nutr..

[18] Gieng S.H., Green M.H., Green J.B., Rosales F.J. (2007). Model-based compartmental analysis indicates a reduced mobilization of hepatic vitamin A during inflammation in rats. J. Lipid Res..

[19] Tan L., Wray A.E., Green M.H., Ross A.C. (2014). Retinol kinetics in unsupplemented and vitamin A-retinoic acid supplemented neonatal rats: A preliminary model. J. Lipid Res..

[20] Tan L., Green M.H., Ross A.C. (2015). Vitamin A kinetics in neonatal rats vs. adult rats: Comparisons from model-based compartmental analysis. J. Nutr..

[21] Hodges J.K., Tan L., Green M.H., Ross A.C. (2017). Vitamin A supplementation redirects the flow of retinyl esters from peripheral to central organs of neonatal rats raised under vitamin A-marginal conditions. Am. J. Clin. Nutr..

[22] Ford J.L., Green J.B., Green M.H. (2019). Addition of vitamin A intake data during compartmental modeling of retinol kinetics in theoretical humans leads to accurate prediction of vitamin A total body stores and kinetic parameters in studies of reasonable duration. J. Nutr..

[23] Green M.H., Ford J.L., Green J.B. (2019). Inclusion of vitamin A intake data provides improved compartmental model-derived estimates of vitamin A total body stores and disposal rate in older adults. J. Nutr..

[24] Ford J.L., Green J.B., Haskell M.J., Ahmad S.M., Mazariegos Cordero D.I., Oxley A., Engle-Stone R., Lietz G., Green M.H. (2020). Use of model-based compartmental analysis and a super-child design to study whole-body retinol kinetics and vitamin A total body stores in children from 3 lower-income countries. J. Nutr..

[25] Li Y., Wei C.H., Green M.H., Ross A.C. (2020). Dietary iron repletion stimulates hepatic mobilization of vitamin A in previously iron-deficient rats as determined by model-based compartmental analysis. J. Nutr..

[26] Kelley S.K., Nilsson C.B., Green M.H., Green J.B., Hakansson H. (2000). Mobilization of vitamin A stores in rats after administration of 2,3,7,8-tetrachlorodibenzo-p-dioxin: A kinetic analysis. Toxicol. Sci..

[27] Ross A.C., Ambalavanan N., Zolfaghari R., Li N.Q. (2006). Vitamin A combined with retinoic acid increases retinol uptake and lung retinyl ester formation in a synergistic manner in neonatal rats. J. Lipid Res..

[28] Li Y., Wei C.H., Xiao X., Green M.H., Ross A.C. (2020). Perturbed vitamin A status induced by iron deficiency is corrected by iron repletion in rats with pre-existing iron deficiency. J. Nutr..

[29] Cifelli C.J., Green J.B., Green M.H. (2007). Use of model-based compartmental analysis to study vitamin A kinetics and metabolism. Vitam. Horm..

[30] Green M.H., Green J.B., Akohoue S.A., Kelley S.K. (2001). Vitamin A intake affects the contribution of chylomicrons vs. retinol-binding protein to milk vitamin A in lactating rats. J. Nutr..

[31] Blomhoff R., Helgerud P., Rasmussen M., Berg T., Norum K.R. (1982). In vivo uptake of chylomicron [^3^H]retinyl ester by rat liver: Evidence for retinol transfer from parenchymal to nonparenchymal cells. Proc. Natl. Acad. Sci. USA.

[32] Ross A.C. (2003). Retinoid production and catabolism: Role of diet in regulating retinol esterification and retinoic Acid oxidation. J. Nutr..

[33] Ross A.C., Zolfaghari R. (2004). Regulation of hepatic retinol metabolism: Perspectives from studies on vitamin A status. J. Nutr..

[34] Sklan D., Ross A.C. (1987). Synthesis of retinol-binding protein and transthyretin in yolk sac and fetus in the rat. J. Nutr..

[35] Ross A.C. (1982). Retinol esterification by mammary gland microsomes from the lactating rat. J. Lipid Res..

[36] Ross A.C., Pasatiempo A.M., Green M.H. (2004). Chylomicron margination, lipolysis, and vitamin a uptake in the lactating rat mammary gland: Implications for milk retinoid content. Exp. Biol. Med..

[37] Akohoue S.A., Green J.B., Green M.H. (2006). Dietary vitamin A has both chronic and acute effects on vitamin A indices in lactating rats and their offspring. J. Nutr..

