# Supplementary material for: Pregnancy and Lactation Alter Vitamin A Metabolism and Kinetics in Rats under Vitamin A-Adequate Dietary Conditions
Source: Nutrients. 2021 Aug 19;13(8):2853. doi: 10.3390/nu13082853 (PMC8401525; doi:10.3390/nu13082853)
Supplement: Supplementary file 1 [file nutrients-13-02853-s001.zip › nutrients-1327665-Supplementary.pdf]

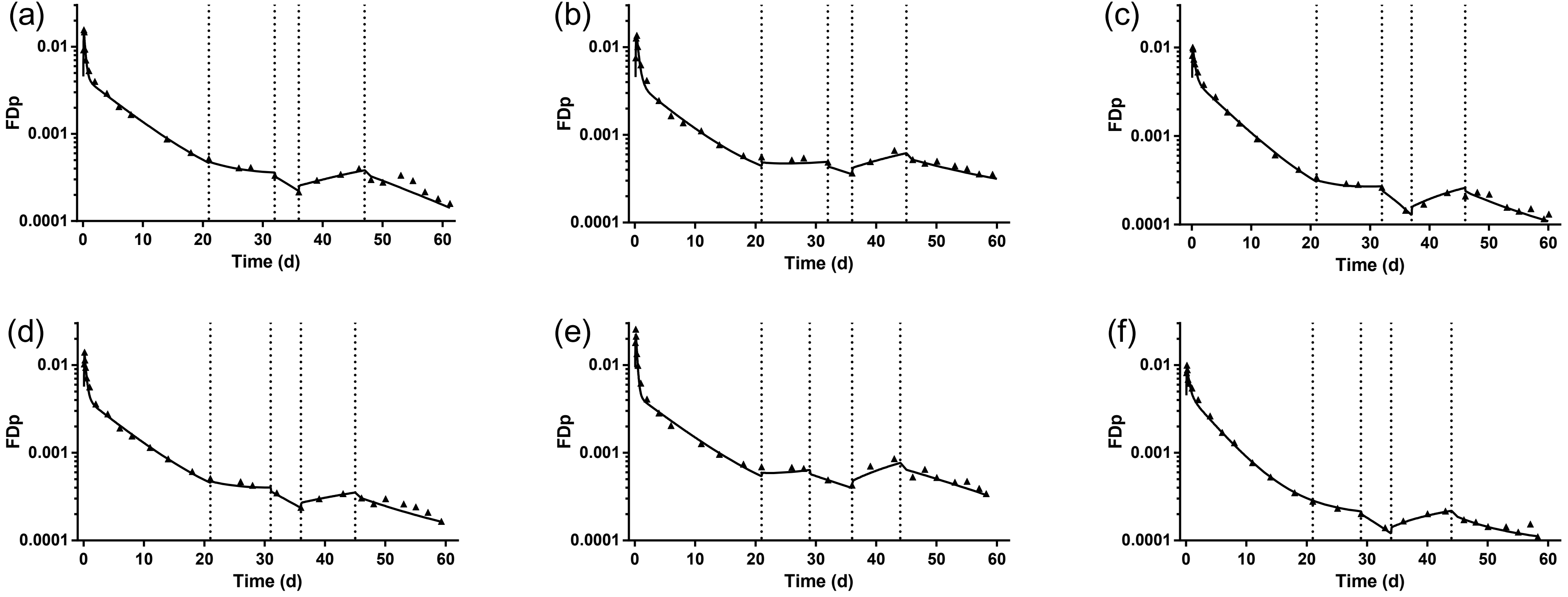

Figure S1: Fraction of administrated dose in plasma versus time (d) after oral delivery of ^{3}H-labeled retinol in pregnant-lactating rats. (a) Rat 1; (b) Rat 2; (c) Rat 3; (d) Rat 4; (e) Rat 5; (f) Rat 6. Solid triangles are observed data; the solid line represents the model-predicted fit; the dashed line indicates the times of perturbation introduced in modeling: the first dashed line (d 21) represents the beginning of mating, the second dashed line (varies by rats) represents early pregnancy, the third dash line (varies by rats) represents late pregnancy, and the fourth dashed line (varies by rats) represents the beginning of lactation. Rat 4 is the represnetative rat.
